# Recurrent Ulnar Nerve Schwannoma in the Cubital Tunnel Elbow: A Rare Presentation and Surgical Management

**DOI:** 10.7759/cureus.73631

**Published:** 2024-11-13

**Authors:** Pankaj Kabra, Mende Vikram Kumar Yadav, Shravan Peddamadyam, Sai Praneeth Bathineedi, Kovuri Yamini

**Affiliations:** 1 Department of Orthopaedics, Nizam's Institute of Medical Sciences, Hyderabad, IND; 2 Department of Orthopaedics, Kamineni Institute of Medical Sciences, Narketpally, IND; 3 College of Medicine, Kamineni Institute of Medical Sciences, Narketpally, IND

**Keywords:** complications, diagnostic imaging, intraneural fibrosis, long-term follow-up, recurrence factors, recurrent schwannoma, surgical difficulties, surgical excision, ulnar nerve

## Abstract

Schwannoma is a benign tumor arising from Schwann cells of peripheral nerves. Although recurrence is rare, this case report highlights a unique instance of recurrent ulnar nerve schwannoma in a 76-year-old construction worker, emphasizing the complexities of surgical management. The patient presented to our orthopedic clinic with persistent pain and tingling in the medial aspect of his left forearm and hand for the past two years. His medical history included bilateral ulnar nerve schwannoma excision at the cubital tunnel level 20 years prior. Examination revealed a firm, non-tender swelling measuring 5 × 5 cm on the medial side of the left distal arm, with neurological assessment indicating reduced sensation in the little finger and medial half of the ring finger, as well as intrinsic hand muscle weakness. His preoperative Disabilities of the Arm, Shoulder, and Hand (DASH) score was 65, reflecting substantial functional limitations. Preoperative nerve conduction studies confirmed ulnar nerve damage, and MRI indicated a tumor originating from the left ulnar nerve. Given the tumor's increasing size, surgical excision was done. The procedure involved careful dissection around the elbow to isolate the ulnar nerve, significantly affected by fibrosis from prior surgeries. We performed macro neurolysis, which decompresses larger segments of the nerve, and micro neurolysis, which allows for precise intervention on specific segments, to effectively address the challenges presented by the scar tissue. Anterior transposition of the ulnar nerve was conducted to place it in an unscarred area, reducing compression and promoting nerve function. The postoperative biopsy confirmed a benign schwannoma characterized by localized Antoni A and Antoni B areas. Six months post surgery, the patient reported complete resolution of symptoms, with grip strength improving to approximately 95% of normal and a postoperative DASH score of 25. Follow-up assessments showed enhanced nerve function, with no signs of tumor recurrence over two years. This case underscores the challenges of managing recurrent ulnar nerve schwannoma and emphasizes the critical role of surgical intervention in preserving nerve function and improving patient outcomes. The successful management of this recurrence highlights the importance of meticulous surgical technique and thorough follow-up in ensuring long-term patient well-being.

## Introduction

Schwannomas, also known as neurilemmomas, are benign tumors that arise from Schwann cells surrounding peripheral, cranial, or autonomic nerves [1]. These tumors occur equally among both sexes, with a peak incidence between the ages of 30 and 40 years [2]. The annual incidence of peripheral schwannomas is approximately 0.6 per 100,000 individuals and they are predominantly found on the flexor surfaces of the limbs [3]. They account for roughly 5% of benign soft tissue neoplasms [4]. Although malignant transformation is rare, distinguishing schwannomas from malignant peripheral nerve sheath tumors can present diagnostic challenges [5].

Ulnar nerve schwannomas, in particular, can lead to nerve compression, manifesting symptoms such as fatigue, discomfort, and pain. This compression disrupts axonal transport, resulting in nerve dysfunction and damage [6]. Typically, schwannomas appear as firm, oval masses measuring under 3 cm, characterized by a well-defined capsule and a smooth surface ranging from yellowish-gray to whitish-gray. Early symptoms often include painless swelling, which can progress to pain, paresthesia, hypoesthesia, and motor deficits [7]. Schwannomas may grow up to 4 cm in size before symptoms arise due to nerve compression [8]. Surgical intervention is generally recommended for symptomatic schwannomas, with potential complications including temporary or permanent sensory or motor deficits [9]. Recurrence is rare, occurring in less than 1% of cases, and is usually associated with incomplete tumor removal [10].

Several studies have examined surgical outcomes for schwannomas, but they generally lack specific recurrence information for ulnar nerve cases. For instance, Gosk et al. reported on 44 schwannomas, including some from the ulnar nerve, but did not provide recurrence rates [11]. Galbiatti et al. noted that none of the 20 patients with ulnar nerve tumors experienced recurrence during a 6-month follow-up, which is too short to assess long-term outcomes [12]. Hirai et al. analyzed complications in 149 patients but did not specify recurrence rates for any particular nerve [13]. Similarly, Raj et al. focused on 21 patients and mentioned complications without detailing recurrence rates over a five-year period [14]. Overall, while these studies offer valuable insights into surgical outcomes, they highlight a significant gap in comparative data and recurrence rates for ulnar nerve schwannomas. Further literature is needed to address these gaps comprehensively.

Histologically, schwannomas are recognized as well-circumscribed masses containing compact spindled areas (Antoni A) and hypocellular, microcystic regions (Antoni B) that include macrophages and collagen fibers. A defining characteristic of schwannomas is their well-formed collagenous capsule and hyalinized vessels. Immunohistochemically, these tumors exhibit strong, diffuse expression of S100 protein and abundant collagen type IV, indicating a continuous basal lamina. Schwannomas may also express glial fibrillary acidic protein (GFAP) and newer markers such as podoplanin, calretinin, and SOX10. Unlike neurofibromas, neurofilament protein staining in schwannomas is generally limited to entrapped axons at the tumor's periphery, although recent studies suggest an increased frequency of intralesional axons [15].

Furthermore, intraneural and perineural fibrosis, often resulting from previous surgeries, can impair nerve function. Perineural fibrosis can elevate nerve tension and lead to ischemia, contributing to pain at rest and during movement, while intraneural fibrosis can result in axonal degeneration and painful neuromas. The primary treatment for schwannomas is surgical excision, emphasizing complete resection to preserve nerve function. Addressing fibrotic changes is essential for symptom relief and restoration of nerve functionality. Nerve tethering due to surgical scars is a significant cause of symptoms linked to perineural scarring, which impairs nerve gliding and exacerbates conditions such as a positive Tinel's sign, hyperalgesia, or allodynia. Imaging modalities like MRI and ultrasound are crucial for preoperative planning and assessing residual or recurrent issues, while electromyography is vital for evaluating nerve distress and monitoring changes over time. Surgical interventions typically involve identifying and mobilizing nerve segments above and below the injury site to ensure they are free from scarring and tethering, then relocating the nerve to a vascularized bed to facilitate gliding [16].

## Case presentation

A 76-year-old construction worker presented to our orthopedic clinic with persistent pain and tingling sensation in the medial aspect of his left forearm and hand. He had experienced these symptoms for the past two years. His medical history included bilateral ulnar nerve schwannoma excision at the cubital tunnel level 20 years prior. On examination, we noted a firm, non-tender, globular swelling measuring 5 × 5 cm on the medial side of his left distal arm. Neurological assessment revealed reduced sensation in the little finger and the medial half of the ring finger. The patient also exhibited weakness in the intrinsic hand muscles, which significantly affected his grip strength, measured at 80% of normal. He reported considerable difficulties with daily tasks such as buttoning his shirt, typing, and securely holding tools, leading him to rely on coworkers for assistance.

**Figure 1:**
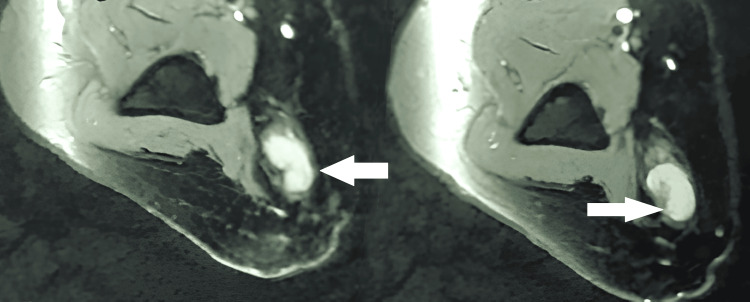
Magnetic resonance imaging (oblique-axial view) demonstrates a mass originating from the left ulnar nerve at the elbow

**Figure 2:**
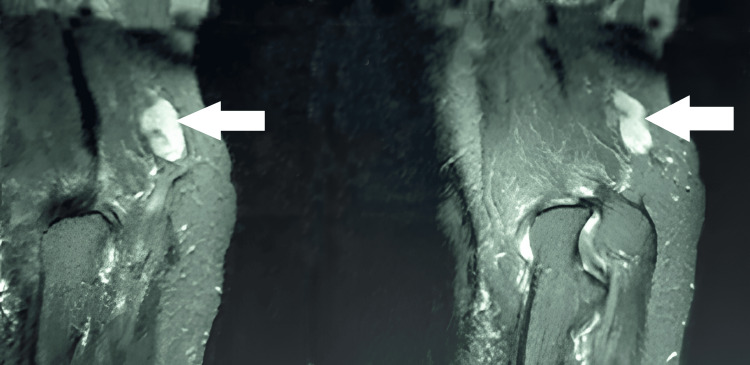
Magnetic resonance imaging (sagittal view) reveals a mass arising from the left ulnar nerve at the elbow

His preoperative Disabilities of the Arm, Shoulder, and Hand (DASH) score was 65, indicating substantial functional limitations that impacted his quality of life and work performance. Preoperative nerve conduction studies confirmed ulnar nerve damage, showing delayed conduction velocities and reduced amplitudes of sensory and motor action potentials. Magnetic resonance imaging (MRI) revealed a well-defined, lobulated T2 hyperintense lesion with homogeneous post-contrast enhancement, seen in the subcutaneous plane along the course of the ulnar nerve, just proximal to the cubital tunnel (Figures 1, 2). There was no evidence of infiltration into deeper soft tissues.

**Figure 3:**
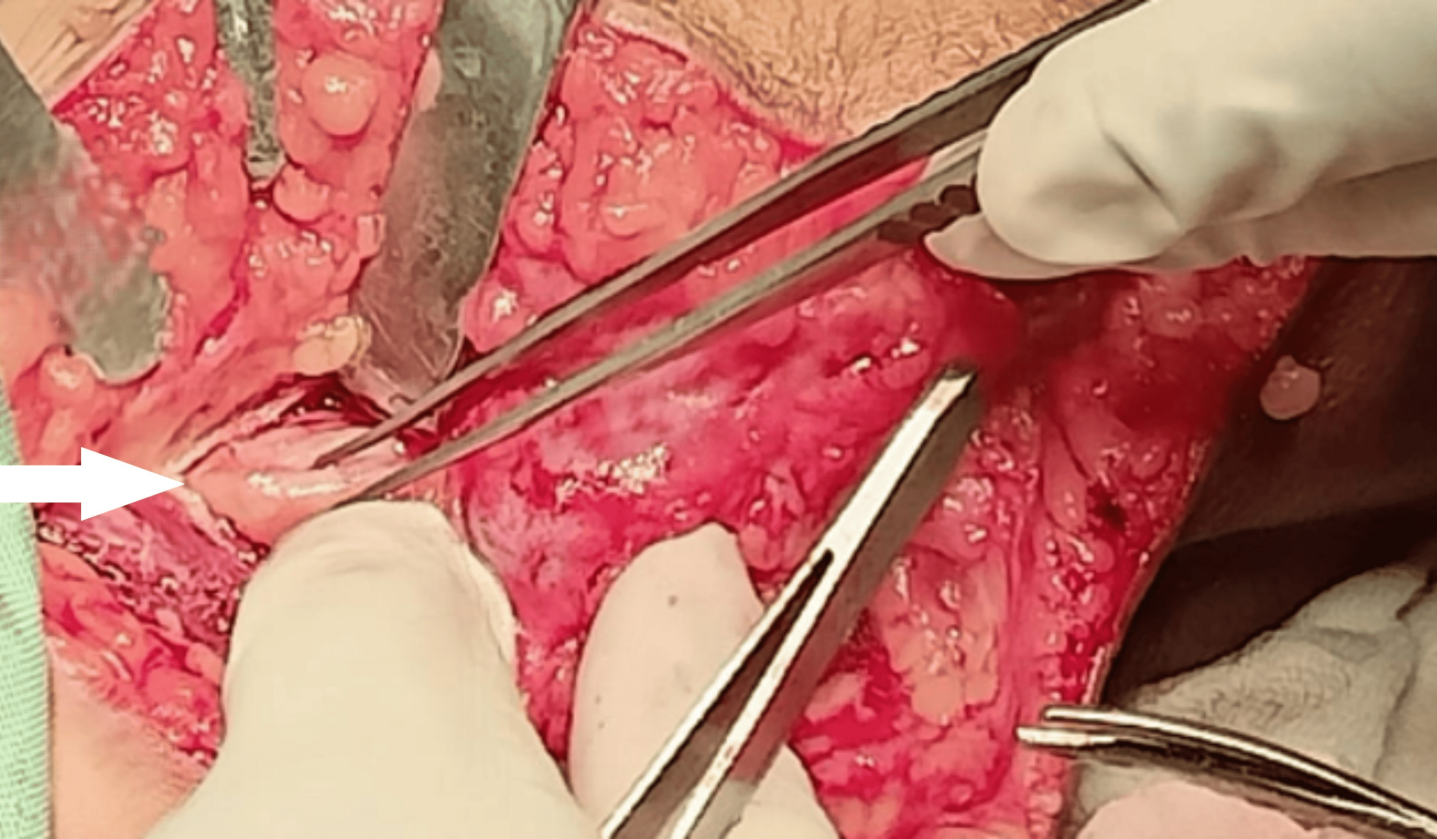
Intraoperative image of the edematous left ulnar nerve following the division of the medial intermuscular septum and the arcade of Struthers

Due to the tumor's increasing size, we performed surgical excision of the ulnar nerve schwannoma. The procedure involved careful medial dissection around the elbow to isolate the ulnar nerve from the distal one-third of the arm to the proximal forearm. We observed significant fibrosis around the nerve from the previous surgery. We released the arcade of Struthers and excised the thickened medial intermuscular septum. The ulnar nerve appeared edematous and tethered (Figure 3). 

**Figure 4:**
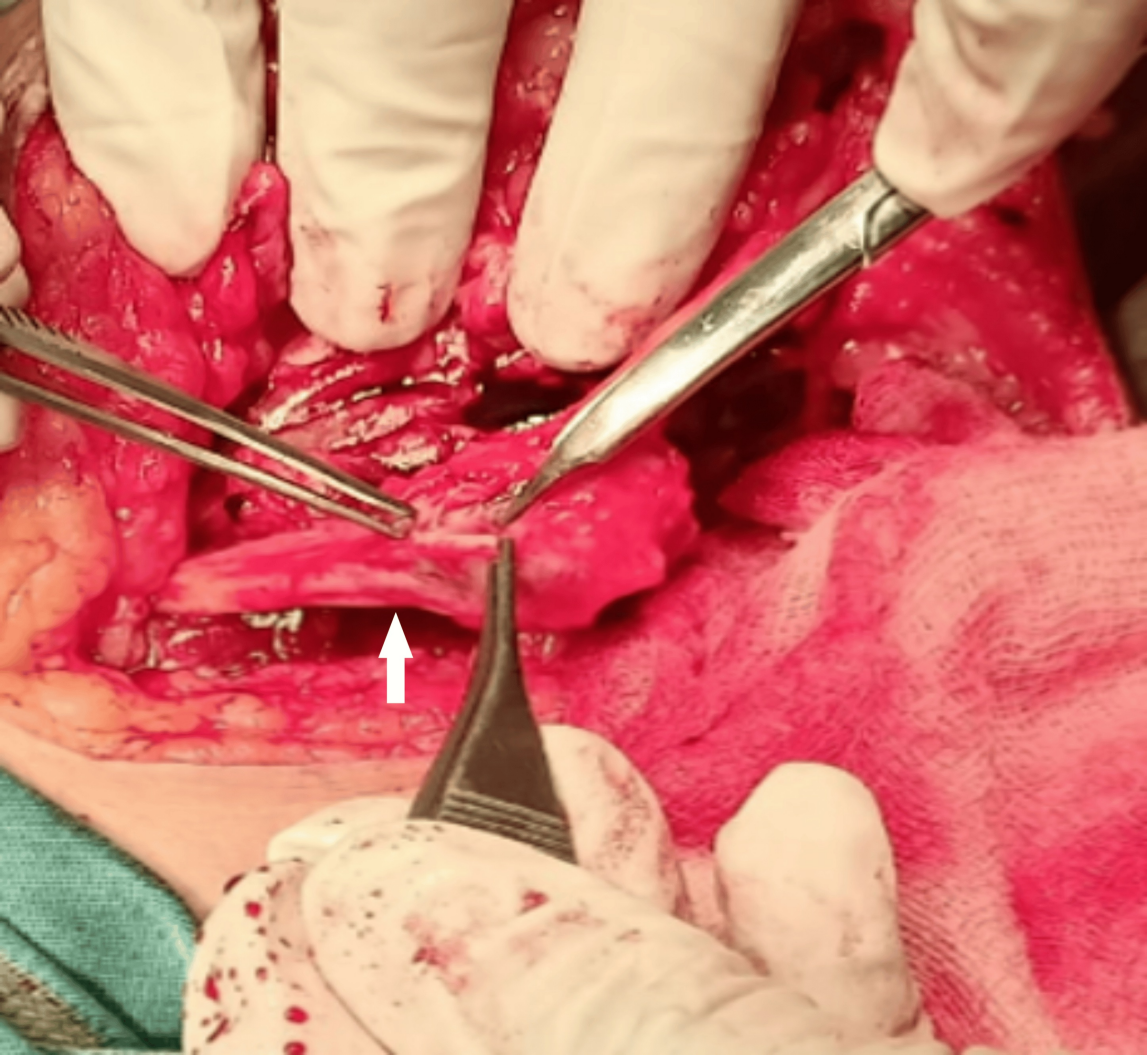
Intraoperative image of tumor excision from the left ulnar nerve

**Figure 5:**
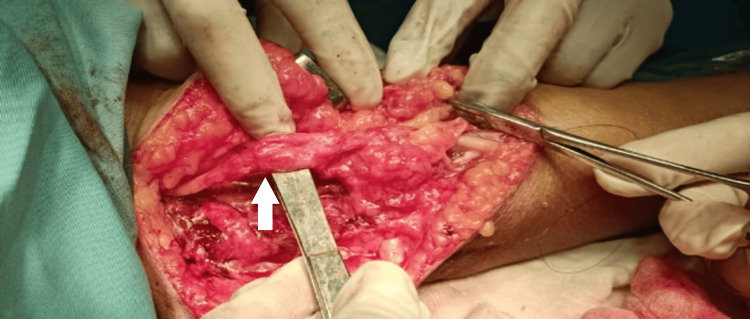
Intraoperative image of tumor excision from the left ulnar nerve and anterior transposition of the left ulnar nerve

**Figure 6:**
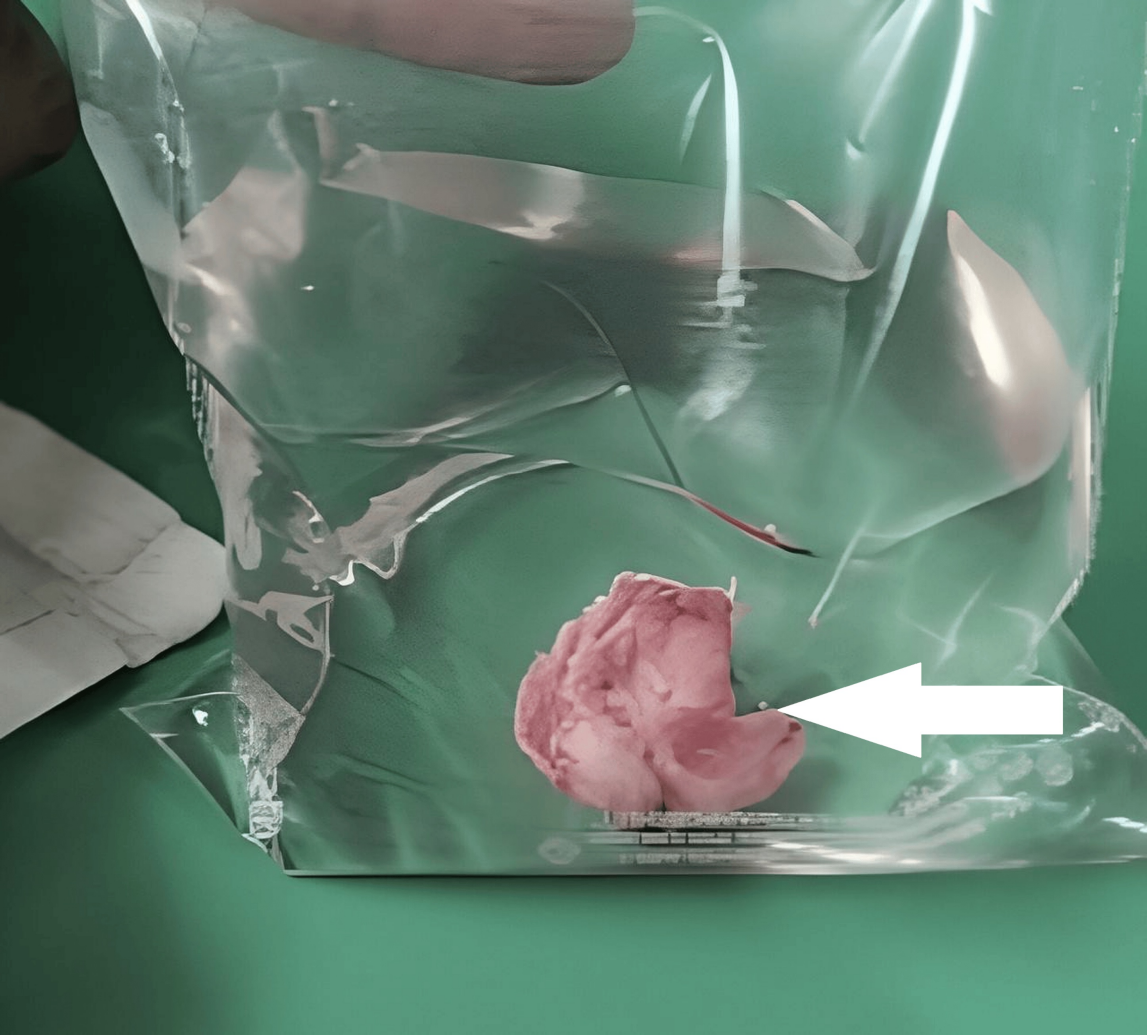
Image showing excised specimen

We also released the Osborne fascia, the two heads of the flexor carpi ulnaris, and the flexor digitorum superficialis origin over the ulnar nerve. Performing macro neurolysis and micro neurolysis proved challenging due to perineural and intraneural fibrosis. We conducted an anterior transposition of the ulnar nerve to place it in an unscarred bed, minimizing further compression. Finally, we excised the schwannoma, including the involved fascicle, while carefully isolating the nerve (Figures 4-6).

We selected anterior transposition of the ulnar nerve for its effectiveness in managing dynamic compression, particularly in patients with significant fibrosis. This technique alleviated strain on the nerve during elbow flexion, which was critical for individuals experiencing severe symptoms that simple decompression might not have adequately addressed. We found that alternative surgical methods, such as simple decompression or medial epicondylectomy, did not fully resolve issues related to extensive fibrosis and ulnar nerve subluxation. While simple decompression relieved pressure, it could have left the nerve vulnerable to ongoing strain. Although medial epicondylectomy offered benefits, it carried risks such as elbow instability and postoperative pain. In contrast, we chose anterior transposition because it specifically targeted the dynamic nature of the condition, making it the optimal choice for this patient. This surgery carried risks, including nerve fibrosis, perineural adhesions, vascular compromise, and infection, particularly in recurrent cases. To mitigate these risks, we prioritized preserving the nerve's blood supply and implemented techniques to reduce friction and scarring. This approach aimed to optimize surgical outcomes and minimize the likelihood of permanent sensory or motor deficits.

**Figure 7:**
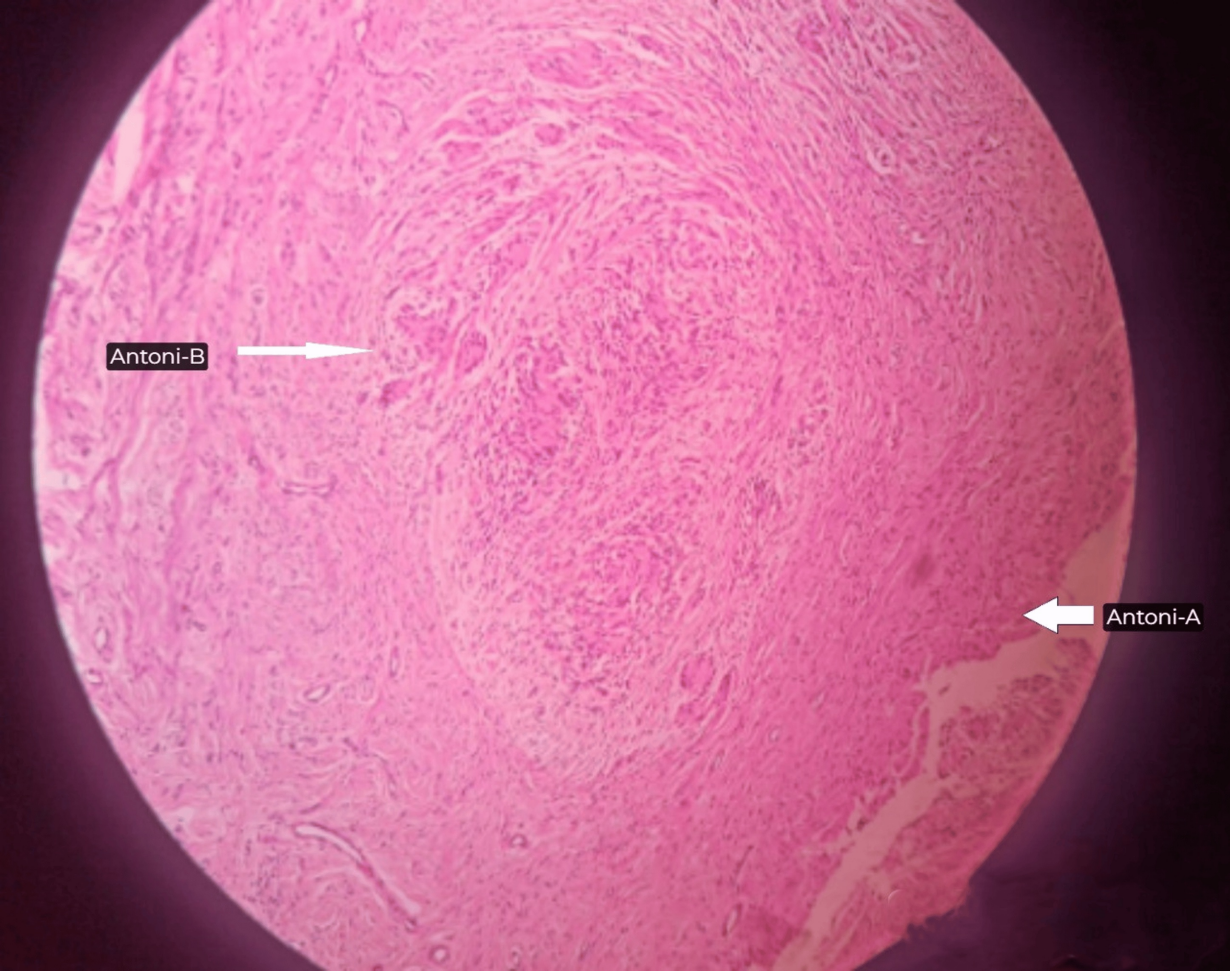
Postoperative biopsy demonstrating a benign schwannoma with focal Antoni A and Antoni B areas

The postoperative biopsy confirmed a benign schwannoma, characterized by localized Antoni A and Antoni B areas (Figure 7). Post surgery, motor and sensory functions were intact (Video 1).

**Video 1:** Video demonstrating preserved motor function at the time of patient discharge

During follow-up, we noted no signs of tumor recurrence, and the patient remained asymptomatic. Six months post surgery, the patient reported complete resolution of paresthesia in the medial aspect of his forearm and hand. Grip strength assessments showed significant improvement. Prior to surgery, his grip strength was measured at 80% of normal, and by six months after the procedure, his grip strength increased to approximately 95% of normal, demonstrating a remarkable recovery. This substantial improvement underscores the effectiveness of the surgical intervention. His postoperative DASH score improved to 25, reflecting considerable enhancements in daily activities and enabling him to perform essential tasks independently. A postoperative nerve conduction study conducted six months after surgery demonstrated improved ulnar nerve conduction velocities and increased amplitudes of both sensory and motor action potentials, indicating enhanced nerve function. The patient continued to experience significant improvements in daily activities. We did not conduct a postoperative MRI because the patient decided against further imaging due to financial concerns and anxiety about the procedure.

Two years post surgery, the patient remained active with no signs of tumor recurrence. His DASH score further improved to 10, demonstrating sustained recovery and the ability to engage fully in work and daily activities without limitations. Regular follow-up appointments confirmed that the patient has remained tumor-free for over two years, with routine assessments every six months to monitor his condition. The long-term prognosis appears encouraging due to the benign nature of the schwannoma and the success of the surgical intervention. Regular follow-up appointments will be essential to monitor for any potential complications, including sensory or motor deficits, and to ensure continued nerve health. Rehabilitation goals will focus on regaining full grip strength and resuming essential work tasks, supported by a structured program of physical and occupational therapy.

## Discussion

Schwannomas are common soft tissue tumors found in the extremities, categorized as proximal (e.g., located on the brachial plexus and upper arm) or distal (e.g., on the forearm and hand). These tumors originate from nerves covered by Schwann cell sheaths and are encased in a true capsule of epineurium. Schwannomas often affect the ulnar nerve. Patients may discover these tumors incidentally as painless masses, or they may experience symptoms due to compression, including neurogenic pain, local swelling, paresthesia, and motor weakness. A positive Tinel's sign strongly indicates these symptoms [17]. The risk of recurrence remains very low, except after subtotal resections. Recurrences can behave like malignant tumors due to distorted anatomy and adhesions from previous surgeries, making subsequent approaches challenging. Thus, complete removal of the tumor, including its capsule, becomes difficult. Using MRI for diagnosis and microsurgical techniques can help avoid recurrence. Therefore, ensuring complete tumor removal during the initial surgery is crucial [18].

In our case of recurrent ulnar nerve schwannoma, the patient exhibited classic symptoms, including sensory deficits and a noticeable mass. An MRI effectively localized the tumor, demonstrating iso-intensity on T1-weighted images and hyperintensity on T2-weighted images. Our surgical approach included macro-neurolysis, micro-neurolysis, and anterior transposition, following best practices that emphasize careful dissection, especially in light of previous surgical interventions. The histopathological examination confirmed the diagnosis as a benign schwannoma, showcasing both Antoni A and B areas. However, we encountered significant challenges during the procedure, primarily due to extensive scar tissue, which complicated the dissection process. This factor may not be as pronounced in other cases, potentially impacting the ease of surgical intervention. While the patient achieved symptom relief and maintained normal motor and sensory function, the literature indicates a higher incidence of postoperative deficits following similar procedures [19]. Focusing on complete tumor removal was a critical component of our strategy aimed at minimizing the risk of recurrence, an issue that often compounds in cases with incomplete resections.

To differentiate schwannomas from other nerve sheath tumors, especially in recurrent cases, a comprehensive approach using advanced imaging techniques is essential. Diagnostic imaging, including ultrasound and MRI, plays a crucial role in localizing tumors and assessing their relationships with surrounding neurovascular structures. Ultrasound typically reveals well-defined hypoechoic masses, while MRI shows schwannomas as isointense on T1 and hyperintense on T2. Histopathological and immunohistochemical examinations remain the gold standards for diagnosis, focusing on hypercellular (Antoni A) and hypocellular (Antoni B) regions, with positive S100 staining distinguishing schwannomas from neurofibromas. Entities that can mimic peripheral nerve sheath tumors (PNSTs) include (i) posttraumatic neuroma, which shows perineural scarring and lacks significant contrast enhancement, (ii) peripheral nerve lipomatosis, characterized by enlarged fascicles and fibrofatty tissue, (iii) Charcot-Marie-Tooth (CMT) disease, noted for symmetrical nerve enlargement and pseudo masses on T2, and (iv) amyloidosis, revealing unilateral or bilateral nerve enlargement with disrupted fascicles. Synovial or fibrosarcoma appears as heterogeneous, large hemorrhagic masses, while intraneural metastatic disease may show restricted diffusion and early arterial enhancement. Neurofibromas present as fusiform or round masses with characteristic features like the tail sign and fascicular sign, indicating close nerve association. Post-surgical imaging typically reveals T2 hyperintensity and contrast enhancement, with recurrence suggested by new nodular lesions or restricted diffusion. Perineuriomas exhibit low to intermediate signal intensity on T1 and heterogeneous high signal intensity on T2, often allowing for clinical monitoring. Malignant peripheral nerve sheath tumors (MPNSTs) appear irregular with indistinct margins and high T2 signal intensity, lacking target or fascicular signs, and show early arterial enhancement with low minimum ADC values on diffusion tensor imaging. Post-surgical findings may indicate increased MRI signal intensity due to edema or changes from surgery. Benign peripheral nerve sheath tumors (BPNSTs) typically display delayed dynamic enhancement patterns with homogeneous or targetoid static enhancement, while MPNSTs show early arterial enhancement with marked heterogeneity. On T1-weighted MRI, BPNSTs are isointense to muscle, while MPNSTs may be homogeneous or heterogeneous. T2-weighted images show BPNSTs as hyperintense with a target sign, common in schwannomas but rare in neurofibromas, whereas MPNSTs appear hyperintense and heterogeneous without a target or fascicular signs. Diffusion tensor imaging indicates that nerve tracts near BPNSTs are near-normal or partially disrupted, with minimum apparent diffusion coefficient (ADC) values exceeding 1.0-1.1 × 10^-3^ mm^2^/second, while MPNSTs show partial or complete disruption, with ADC values typically below 1.0-1.2 × 10^-3 ^mm^2^/second. Spectroscopy analysis shows the trimethylamine fraction in BPNSTs is generally less than 50%, while in MPNSTs, it often exceeds 50%. Morphologically, BPNSTs are typically round or oval and measure less than 5 cm, whereas neurofibromas demonstrate continuity with the nerve and are encapsulated, and MPNSTs are often irregular or round, frequently exceeding 5 cm. BPNSTs have well-defined margins, while MPNSTs may have either well-defined or ill-defined margins and can exhibit invasion. Necrosis and cystic changes are uncommon in BPNSTs but more frequent in MPNSTs, which also show higher rates of hemorrhage and calcification. Perilesional edema is usually absent in BPNSTs but common in MPNSTs. Imaging characteristics help clinicians differentiate BPNSTs from MPNSTs, which is crucial for diagnosis and treatment planning [20].

In schwannomas, distinguishing between Antoni A and B areas is essential for understanding tumor behavior and the potential for recurrence. Antoni A areas, characterized by compact, palisading spindle cells, often exhibit heightened mitotic activity, particularly in childhood plexiform schwannomas, which frequently lack Antoni B areas. This increased mitotic index can elevate the risk of misdiagnosis as malignant peripheral nerve sheath tumors, particularly those associated with neurofibromatosis type 1. Histopathological features such as brisk mitosis and high proliferation rates correlate with local recurrences in pediatric cases, highlighting the necessity for a thorough evaluation. Immunohistochemical stains, notably S100 positivity, serve as important diagnostic tools that aid in differentiating benign schwannomas from malignant tumors and suggest a more favorable prognosis when pleomorphism, necrosis, and vascular invasion are absent. Therefore, integrating these histopathological markers and immunohistochemical findings is crucial for enhancing our understanding of recurrence risks in schwannomas [21].

Diagnostic imaging techniques, such as ultrasound and MRI, play a pivotal role in accurately diagnosing schwannomas by localizing tumors and assessing their relationships with surrounding neurovascular structures. Although these methods have limitations, they are critical for effective surgical planning. Ultrasound can reveal well-defined hypoechoic masses associated with the nerve, while MRI typically shows schwannomas as isointense on T1 and hyperintense on T2 images. Histopathological and immunohistochemical examinations remain the gold standards for diagnosis, with features such as hypercellular (Antoni A) and hypocellular (Antoni B) regions on histopathological examination and positive S100 staining on immunohistochemical examination helping to differentiate schwannomas from neurofibromas [22].

In the context of vestibular schwannoma, emerging nonsurgical treatments such as stereotactic radiosurgery (SRS) and fractionated stereotactic radiotherapy (FSRT) provide valuable alternatives to surgical intervention, even though they were not used in this specific case. SRS, typically delivered at around 12 Gy, offers high local control rates exceeding 90% while minimizing risks such as facial and trigeminal nerve dysfunction. This approach is particularly beneficial for patients seeking to preserve hearing or for those who are not surgical candidates. FSRT serves as a viable option for managing residual or recurrent tumors, providing effective control with a favorable side effect profile. However, potential risks include temporary side effects and the rare possibility of radiation-induced tumors, such as MPNSTs, which necessitate careful monitoring. Regular follow-up with imaging is essential to assess tumor stability and address any late-onset complications. Additionally, ongoing research into targeted therapies may further enhance treatment options, potentially improving outcomes and reducing recurrence rates [23].

Microsurgical resection is recommended for treating all symptomatic schwannomas at first diagnosis and for asymptomatic cases with MRI evidence of increasing tumor size [24]. Surgical decision-making for schwannomas typically relies on clinical and radiological findings. Since schwannomas comprise neoplastic Schwann cells surrounding the nerve, achieving complete resection may necessitate excising tumor-invaded nerve tissue. This surgical approach sometimes risks permanent or transient sensory or motor deficits. Therefore, it is essential to carefully consider the tumor's morphology, its topographical location, and the patient's residual nerve function. This comprehensive evaluation helps balance the goals of complete resection with the potential for nerve damage [25]. In the follow-up phase, MRI and regular clinical examinations monitor tumor recurrence and functional improvement. Complete excision is recommended initially to reduce the risk of schwannoma recurrence, emphasizing the importance of correct surgical technique despite potential difficulties [26]. Schwannomas can often be excised with minimal damage to nerve fascicles due to their eccentric, noninfiltrating growth [27]. However, the possibility of developing new postoperative neurological deficits remains. Factors contributing to this include potential damage to fascicles encircling the tumor during epineural incision and irritation or compression of healthy fascicles during dissection.

Park et al. suggest that transecting fascicles entering the tumor may lead to deficits [28], while Donner et al. argue that these fascicles are usually nonfunctional, and their transection does not cause additional issues [29]. Although schwannomas rarely induce motor function impairment, any motor deficit raises suspicion of malignancy [30]. Extracapsular excision is a common technique for schwannoma removal, but modifications can reduce nerve fascicle damage. Hussain et al. recommend incising the capsule laterally and dissecting the tumor circumferentially while leaving the epineural capsule intact for protection [31]. Date et al. found the intracapsular technique superior due to lower complication rates [32]. Microenucleation, which involves removing the tumor piece by piece, may be employed in cases with adhesions between the epineurium and capsule. Finally, while schwannomas grow slowly, allowing for some adaptation in nerve function, they eventually become symptomatic, necessitating surgical intervention [33]. Even with careful dissection of the schwannoma from the involved nerve under magnification, neurological deficits can still occur. The transection of fascicles running through the tumor is believed to be the main cause of postoperative deficits. Sawada et al. hypothesized that longitudinal incisions in the nerve sheath may divide a few taut fascicles over the tumor mass [34]. Additionally, they found a significant relationship between a positive preoperative Tinel's sign and neurological complications. Takase et al. noted that damage to fascicles during dissection is a primary cause of new postoperative deficits and the risk of developing neurological deficits is higher in patients with larger tumors [35]. Conversely, Oberle et al. reported that a longer symptom history is associated with postoperative deficits [36].

## Conclusions

Effective management of schwannomas is crucial for minimizing recurrence risk and preserving neural function. Surgical excision requires meticulous techniques, as previous surgeries can complicate tumor resection and increase postoperative neurological morbidity, as demonstrated in this case of recurrent ulnar nerve schwannoma. High-resolution imaging, particularly MRI, plays a vital role in precise tumor localization and surgical planning, leading to improved patient outcomes. Employing microsurgical techniques, such as macro- and micro-neurolysis, is essential for carefully addressing fibrosis while preserving surrounding neural structures and optimizing functional recovery. Histopathological confirmation is critical for distinguishing schwannomas from other peripheral nerve tumors and guiding effective management.

Regular follow-up and imaging are essential for the early detection of potential recurrence and for assessing functional recovery, with tumor size and symptom duration serving as significant predictors of complications. Therefore, obtaining informed consent and engaging in thorough discussions about treatment options, risks, and benefits is necessary. Future research should focus on advanced imaging modalities, minimally invasive surgical techniques, and novel therapeutics to enhance the management of schwannomas. By emphasizing early detection and complete excision, along with adhering to these principles, multidisciplinary teams can provide optimal care for patients with schwannomas and significantly improve long-term outcomes.
